# Optogenetic approaches for functional mouse brain mapping

**DOI:** 10.3389/fnins.2013.00054

**Published:** 2013-04-10

**Authors:** Diana H. Lim, Jeffrey LeDue, Majid H. Mohajerani, Matthieu P. Vanni, Timothy H. Murphy

**Affiliations:** ^1^Department of Psychiatry, University of British Columbia at VancouverVancouver, BC, Canada; ^2^Brain Research Centre, University of British Columbia at VancouverVancouver, BC, Canada

**Keywords:** optogenetic stimulation, Channelrhodopsin-2, *in vivo* imaging, functional mapping, connectivity

## Abstract

To better understand the connectivity of the brain, it is important to map both structural and functional connections between neurons and cortical regions. In recent years, a set of optogenetic tools have been developed that permit selective manipulation and investigation of neural systems. These tools have enabled the mapping of functional connections between stimulated cortical targets and other brain regions. Advantages of the approach include the ability to arbitrarily stimulate brain regions that express opsins, allowing for brain mapping independent of behavior or sensory processing. The ability of opsins to be rapidly and locally activated allows for investigation of connectivity with spatial resolution on the order of single neurons and temporal resolution on the order of milliseconds. Optogenetic methods for functional mapping have been applied in experiments ranging from *in vitro* investigation of microcircuits, to *in vivo* probing of inter-regional cortical connections, to examination of global connections within the whole brain. We review recently developed functional mapping methods that use optogenetic single-point stimulation in the rodent brain and employ cellular electrophysiology, evoked motor movements, voltage sensitive dyes (VSDs), calcium indicators, or functional magnetic resonance imaging (fMRI) to assess activity. In particular we highlight results using red-shifted organic VSDs that permit high temporal resolution imaging in a manner spectrally separated from Channelrhodopsin-2 (ChR2) activation. VSD maps stimulated by ChR2 were dependent on intracortical synaptic activity and were able to reflect circuits used for sensory processing. Although the methods reviewed are powerful, challenges remain with respect to finding approaches that permit selective high temporal resolution assessment of stimulated activity in animals that can be followed longitudinally.

## Introduction

One of the major goals in neuroscience has been to map the connectivity of the brain. From the classical structural studies by Ramón y Cajal, which implied connections between pairs of neurons (Swanson and Swanson, 1995), to the more recent endeavor to reverse-engineer the human brain (Markram, 2006), it is clear that understanding the brain's connections has been long sought-after (Toga and Thompson, 2003; Sporns et al., 2004; Lichtman and Denk, 2011; Seung, 2011). Despite this, generating connectivity maps has proven to be challenging. In fact, the only nervous system that has been comprehensively mapped at a structural level is from the nematode, *C. elegans* (White et al., 1986), and while progress has been made in generating neuronal connectivity maps in the mouse (Mayerich et al., 2008; Bohland et al., 2009; Li et al., 2010; Lichtman and Denk, 2011; Allen Institute for Brain Science, 2012; Denk et al., 2012), and the rat brain (Zakiewicz et al., 2011), their higher level of complexity is a major barrier, and these maps do not address brain function.

In order to generate a more comprehensive connectivity map of a mammalian system, structure and function will need to be considered and integrated (Lichtman and Denk, 2011; Leergaard et al., 2012). Anatomical studies mapping structural connectivity from Cajal to the present (Lein et al., 2007; Allen Institute for Brain Science, 2012) have been important in shaping our current understanding of how the brain is wired and the organization of structural brain networks (Sporns et al., 2004). Now, with some basic understanding of the structural anatomy of the brain, experiments investigating functional connectivity are necessary to determine how the brain operates within its structural framework. In the case of functional connectivity, there will not be a single map. Behavioral state will affect apparent connections (Kohn et al., 2009), as observed in mice (Petersen et al., 2003; Crochet and Petersen, 2006) and rats (Erchova et al., 2002) under various levels of anesthesia. Thus, both structural maps indicating possible neuronal pathways, and functional maps indicating the strength of connection between neurons within specific areas, will be required to better appreciate brain connectivity.

One tool that has been recently developed and applied for cortical mapping is optogenetics (Nagel et al., 2003; Boyden et al., 2005; Fenno et al., 2011). Optogenetics uses a combination of light and genetic manipulation in order to control a biological system. There are a number of different recombinant proteins that can be introduced into neural systems and used for this purpose. One of the proteins that has had the most significant use in neuroscience is Channelrhodopsin-2 (ChR2), which is a non-selective cation channel that opens in response to blue light (Nagel et al., 2003). In mammalian systems, expression of ChR2 or other opsins have been achieved through a number of different means (for a review of neuronal labeling, see Young and Feng, 2004). These include local injection of a viral vector (Zhang et al., 2006; Kuhlman and Huang, 2008), *in utero* electroporation of a genetic construct (Petreanu et al., 2007; Huber et al., 2008), or the creation of transgenic mouse lines (Arenkiel et al., 2007; Madisen et al., 2012; Ting and Feng, 2013). Care must be taken in designing optogenetic experiments because ChR2 stimulation may evoke relatively larger calcium transients (compared to current injection), that may lead to an increased probability of neurotransmitter release, which could lead to plastic changes within a neuronal circuit (Schoenenberger et al., 2010), and because over-expression of ChR2 may have an effect on neuronal function and viability (Miyashita et al., 2013). Despite these potential drawbacks, since the introduction of optogenetics in neuroscience in 2005 (Boyden et al., 2005), there has been an explosion of studies utilizing this tool (Fenno et al., 2011; Yizhar et al., 2011a). ChR2 and other opsins have been applied in studies at various levels of neuronal organization, extending from *in vitro* studies using brain slices (Mateo et al., 2011; Avermann et al., 2012) to *in vivo* studies using awake, transgenic animals (Huber et al., 2008; Desai et al., 2010; Poulet et al., 2012).

In this review, we will address functional mapping techniques that use ChR2 (Nagel et al., 2003, 2005; Boyden et al., 2005) to interrogate cortical activity, as well as their respective advantages and disadvantages (Table 1). Although methods using alternative opsins, including inhibitory opsins such as Halorhodopsin (Han and Boyden, 2007) and Archeorhodopsin (Chow et al., 2010) have been developed, we will focus on methods that use ChR2. Furthermore, while methods exist for mapping cortical connectivity in a number of different animal models (Nagel et al., 2005; Diester et al., 2011), we will focus on techniques used for mapping connectivity in the rodent brain, where recent progress in the creation of transgenic lines (Arenkiel et al., 2007; Madisen et al., 2010, 2012; Chen et al., 2012a; Zeng and Madisen, 2012; Ting and Feng, 2013) is creating remarkable opportunities for investigating brain function.

**Table 1:** **Optogenetic functional mapping techniques**.

**Technique**	**Spatial and temporal resolution**	**Advantages**	**Limitations**	**Future directions**	**Select references**
Single-cell E.phys and ChR2 stimulation/CRACM	Single synapse or microcircuit (μm) spatial resolution Microsecond (μs) temporal resolution	High temporal resolution Can define layer-specific connections (CRACM)	Sparse spatial sampling Not suitable for chronic studies	Assess response using optical tools (i.e., GECIs)	Petreanu et al., 2007; Mateo et al., 2011; Avermann et al., 2012
ChR2-mediated light-based motor mapping	Regional (<1 mm) spatial resolution Millisecond (ms) temporal resolution	Relatively non-invasive for chronic studies	Motor output, not cortical output measured Limited to motor cortex	Red-shifted opsins to minimize light scattering in tissue Tracking disease progression	Ayling et al., 2009; Hira et al., 2009; Harrison et al., 2012
VSD imaging and ChR2 stimulation *in vivo*	Regional (100 s of μm – mm) spatial resolution Millisecond (ms) temporal resolution	VSD (RH1692) excitation does not activate ChR2 Can map cortical areas independent of behavior or sensory processing	Phototoxicity/not suitable for chronic studies Limited to cortex Non-specific dye targeting Images during photostimulation are saturated	Cell-specific targeting and chronic imaging (i.e., VSFPs—see Akemann et al., 2010)	Lim et al., 2012
Opto-fMRI	Global (mm) spatial resolution	Relatively non-invasive for chronic studies	Relatively undefined BOLD signal	Multiple sites of stimulation	Desai et al., 2010; Lee et al., 2010; Kahn et al., 2011
	Second (s) temporal resolution	Can be done in awake or anesthetized animals	Animals must be head-fixed	Longitudinal studies of disease and/or plasticity	
				Poor temporal resolution		

### Functional mapping of synaptic and columnar architecture using optogenetic stimulation in brain slices

Initially, electrophysiological recordings were used to investigate functional properties of individual and groups of neurons in response to single-point electrical stimulation (Scanziani and Hausser, 2009) or glutamate uncaging (Callaway and Katz, 1993; Katz and Dalva, 1994; Hooks et al., 2011). These techniques offer excellent temporal resolution at the level of the single cell and have been used to create layer-specific wiring diagrams of the rodent sensory and motor cortex (Weiler et al., 2008; Anderson et al., 2010). Although these are labor-intensive experiments, they provide detailed data on synaptic connectivity and the temporal dynamics of the response. Such mapping studies are best applied in brain slices and typically have not evaluated *in vivo* connectivity. These techniques complement emerging high-throughput structural mapping techniques (Denk and Horstmann, 2004; Lu et al., 2009; Kleinfeld et al., 2011; Gong et al., 2013).

**Figure 1:**
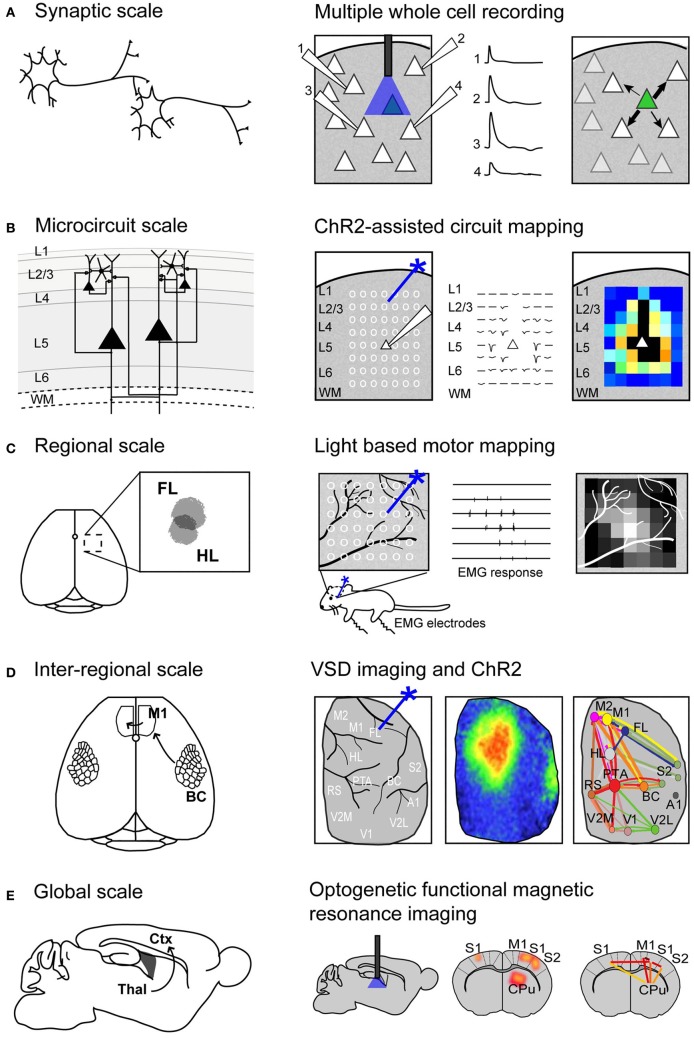
**Using Channelrhodopsin-2 stimulation to map functional connectivity at multiple scales of mouse brain organization. (A)** Mapping functional synaptic connectivity between individual neurons (left) using multiple whole-cell recordings (right). ChR2-expressing neurons (indicated in green) are photostimulated and the electrophysiological response is recorded in one or more target neurons (numbered 1–4). **(B)** Mapping functional connectivity within a column of neurons or a microcircuit (left) using ChR2-assisted circuit mapping (right). Multiple regions within the column are photostimulated (indicated by white circles) and the response is recorded from a single target neuron (white triangle). L, layer; WM, white matter. **(C)** Mapping functional connectivity within a region of cortex (left) using light-based motor mapping (right). A number of regions within the motor cortex are targeted for photostimulation (indicated by white circles) and the resulting motor responses are recorded through electromyograms (EMG) to determine function. FL, forelimb area of the sensorimotor cortex; HL, hindlimb area of the sensorimotor cortex. **(D)** Mapping inter-regional functional connectivity (left) using VSD imaging and ChR2 stimulation (right). Multiple regions of interest are targeted for photostimulation and the resulting VSD response (change in fluorescence) is recorded to indicate strength of connections between functional regions. A1, auditory cortex; BC, barrel cortex; FL, forelimb area of the primary somatosensory cortex; HL, hindlimb area of the primary somatosensory cortex; M1, primary motor cortex; M2, secondary motor cortex; PTA, partial association cortex; RS, retrosplenial cortex; V1, primary visual cortex; V2L, lateral secondary visual cortex; V2M, medial secondary visual cortex. **(E)** Mapping global functional connectivity (left) using optogenetic functional magnetic resonance imaging (right). ChR2-expressing neurons are photostimulated with an optical fiber and the resulting BOLD signal is recorded over the whole brain. CPu, caudate putamen; Ctx, cortex; M1, motor cortex; S1, somatosensory cortex; S2, secondary somatosensory cortex; Thal, thalamus.

Recently, electrophysiological recordings have been applied in combination with optogenetics to investigate cell-type-specific responses within a small network (Figure 1A). Avermann and colleagues investigated synaptic connectivity in the mouse barrel cortex *in vitro* by comparing synaptic responses from different neuronal types (Avermann et al., 2012). Layer 2/3 excitatory neurons expressing ChR2 were photostimulated, and responses were measured from GABAergic fast-spiking (FS) and GABAergic non-fast-spiking (NFS) cells through multiple simultaneous whole-cell recordings. Stimulation of the layer 2/3 neurons resulted in large-amplitude depolarizing postsynaptic potentials in FS GABAergic neurons, but small-amplitude subthreshold postsynaptic potentials in NFS GABAergic neurons. The authors suggest that FS GABAergic neurons play a large role in excitatory neuron inhibition within the barrel cortex. This study demonstrates the use of optogenetic methods in an *in vitro* brain slice preparation and the ability to monitor functional synaptic connectivity.

An important topic in brain connectivity is understanding how neurons can integrate multiple inputs, and the function of a neuron within the larger scope of a circuit. Optogenetic techniques have been applied to questions concerning circuit-level connectivity in mouse brain by targeting multiple sites for photostimulation at the level of the subcellular compartments, such as afferent axons (Petreanu et al., 2009) (Figure 1B). **ChR2-assisted circuit mapping (CRACM)** combines photostimulation of multiple points in the sample with postsynaptic recording to investigate cortical circuits in brain slices (Petreanu et al., 2007, 2009; Atasoy et al., 2008; Haubensak et al., 2010). Similar to LSPS, which maps excitatory connections within a network through glutamate uncaging and postsynaptic recordings (Callaway and Katz, 1993; Katz and Dalva, 1994), CRACM maps excitatory connections through optogenetic stimulation. However, unlike LSPS, which excites all cells but not axons of passage (Katz and Dalva, 1994; Petreanu et al., 2007), CRACM excites only ChR2-expressing cells, allowing for cell-type-specific mapping of local and long-range cortical circuits, such as those found within a cortical column (Petreanu et al., 2007, 2009; Mao et al., 2011; Hooks et al., 2013). For example, using CRACM in the somatosensory cortex of the mouse, layer-specific projections were identified and quantified (Petreanu et al., 2007). Layer 2/3 axons had the strongest connection with layer 5 neurons, followed by layer 2/3 and layer 6 neurons, but not layer 4 neurons. This laminar specificity was similar in ipsilateral (local) and contralateral (callosal) projections. Such microcircuits are presumed to be stereotypical within a certain cortical area and form the foundation of cortical function (Douglas and Martin, 2004), making the investigation of these circuits central to our understanding brain connectivity. In the future, it is expected that photostimulation and cellular optical recording using organic or genetic voltage or calcium sensors (Grinvald and Hildesheim, 2004; Knopfel, 2012) (described in section Functional Regional Mapping using Optogenetic Stimulation *in vivo*) will be combined to allow the recording of signals from large ensembles of cells. Moreover, the recent advance of two-photon-mediated excitation of ChR2 (Papagiakoumou et al., 2010; Packer et al., 2012; Rickgauer and Tank, 2012) may help to produce more discrete activation in future studies.

The CRACM technique is desirable because it can isolate the specific contribution of each cell within the slice, avoiding long-range connections and subcortical contributions. At the same time, however, this technique cannot be used for within-animal longitudinal studies because it is limited to brain slices. Furthermore, while patterns of photostimulation using various sequences and rapid scanning can be used to investigate the basic connectivity within a circuit, it is unlikely that it can effectively mimic the numerous inputs, various sequences of activation, and temporal relationships between multiple neurons that are constantly contributing to the function of the circuit *in vivo* (Katz and Dalva, 1994). For this reason, it is important to consider both *in vitro* and *in vivo* experiments in order to understand functional brain connectivity.

### Functional mapping of the sensorimotor cortex using optogenetic stimulation *in vivo*

Early maps of the human cortex were based on structure and relied on cytoarchitecture to define boundaries between cortical areas (Garey, 2006; Triarhou, 2007). In order to create an organizational map based on function rather than structure, a method is required that has the ability to stimulate the cortex and has a means of measuring the output. Historically, this has been achieved in humans by observing behavioral output following cortical stimulation with single penetrating electrodes (Penfield and Boldrey, 1937; Penfield and Rasmussen, 1950), or in animal models by measuring motor output following intracortical microstimulation (ICMS) at multiple sites of the sensorimotor cortex (Neafsey et al., 1986; Mitz and Wise, 1987).

**Figure 2:**
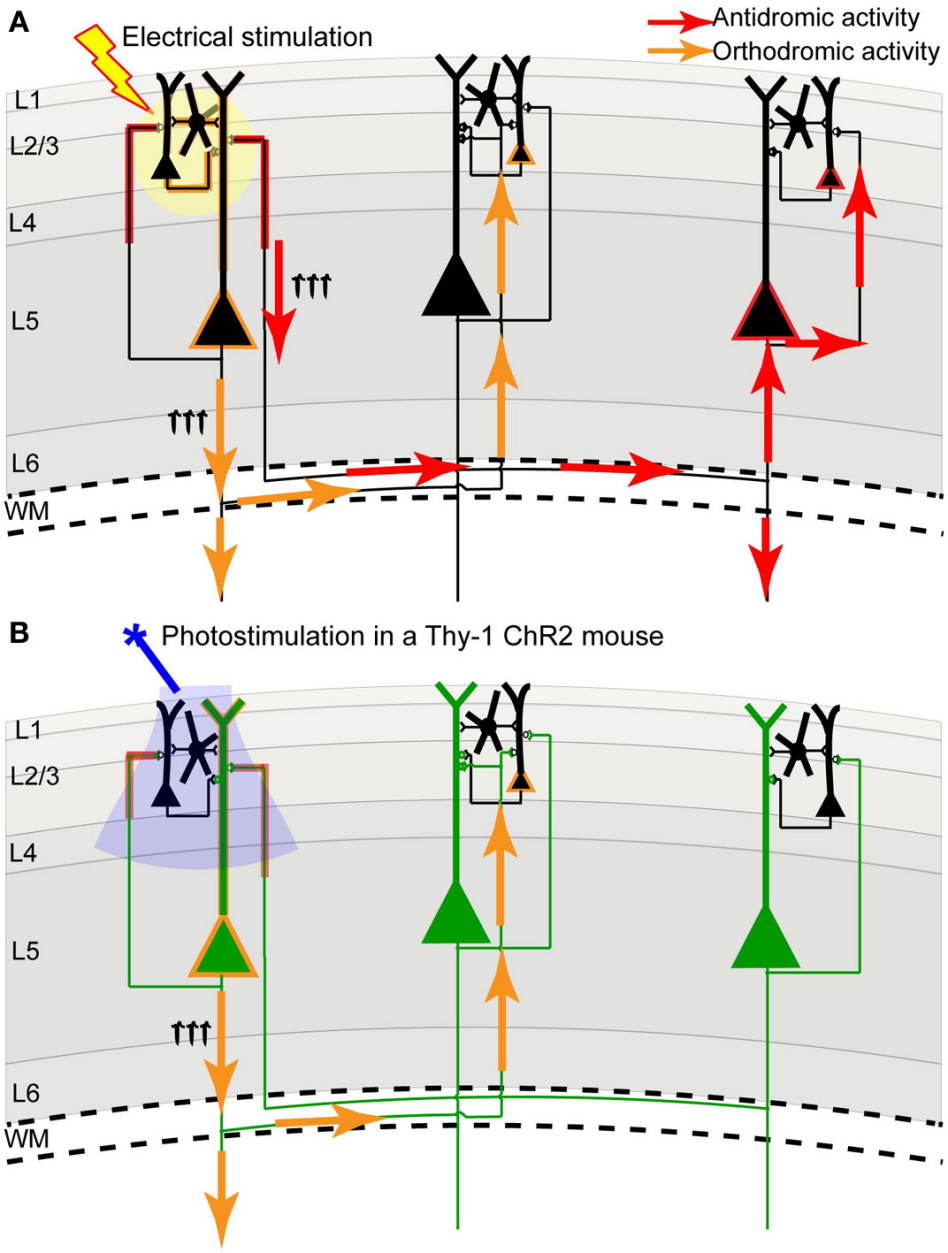
**Channelrhodopsin-2 stimulation in the Thy-1 transgenic mouse may result in more specific activation compared to direct electrical stimulation. (A)** In direct electrical stimulation all cellular components and all cell types within the area of electrical stimulation (indicated by the yellow circle) are activated, inducing both antidromic (axon to soma/dendrite; red arrows) and orthodromic (dendrite/soma to axon; orange arrows) activation. Because electrical stimulation can activate axons (including axons of passage), it may reveal the source of a projection and not its target. **(B)** In photostimulation of the Thy-1 line 18 transgenic mouse (Arenkiel et al., 2007), predominantly layer 5 ChR2-expressing neurons (indicated in green) are directly activated. While ChR2 is expressed in both axons and dendrites, we suggest that the tuft dendrites of layer 5 neurons are more prominent near the brain surface (where light stimulation is directed) and more likely to become activated, inducing primarily orthodromic synaptic stimulation of distant targets (as determined by antagonist sensitivity; Lim et al., 2012).

We and others have developed a new method—**light-based mapping (LBM)**—for *in vivo* sensorimotor cortex mapping in the mouse (Ayling et al., 2009; Hira et al., 2009; Komiyama et al., 2010). This method uses optogenetics to stimulate the sensorimotor cortex with high spatiotemporal resolution while recording-evoked motor movements to determine cortical function (Figure 1C). A grid of stimulation points was targeted over the sensorimotor cortex of transgenic mice (line 18, from Jackson labs) predominantly expressing ChR2 in the layer 5 pyramidal cells in the cortex (Arenkiel et al., 2007). Sites were photostimulated with 473 nm light to selectively activate ChR2-expressing cells, and the evoked motor responses (electromyograms) were recorded. ICMS can be used in a similar fashion to map the topography of the motor cortex (Neafsey et al., 1986; Mitz and Wise, 1987), however, electrical stimulation will activate all cells and axons of passage without discrimination (Asanuma et al., 1976) and will stimulate both orthrodromic and antridromic activity (Tehovnik, 1996; Histed et al., 2009; Griffin et al., 2011) (Figure 2A), bringing in to question how well the stimulated activity resembles endogenous cortical activity. Moreover, ICMS is not suitable for longitudinal studies. In contrast, LBM selectively stimulates ChR2-expressing neurons, allowing for more precise control of which connections are being stimulated (Figure 2B). While ICMS can be used to map the sensorimotor cortex with relatively high precision, there is a time-cost involved in raising and lowering the stimulating electrode at each site, and electrode insertion will cause tissue damage and could alter cortical function. In comparison, hundreds of cortical sites can be optically stimulated (in a randomized order), in a matter of minutes with LBM, and the stimulation is less invasive, allowing for repeated mapping over time without the risk of tissue damage associated with ICMS (Harrison et al., 2012). Repeated sampling using LBM may result in maps with fewer artifacts and higher definition. Furthermore, rapid stimulation is important for *in vivo* anesthetized experiments since cortical activity is affected by time-dependent changes in the depth of anesthesia (Erchova et al., 2002).

While LBM has several advantages over ICMS, the spatial resolution using LBM will be limited due to overlapping dendritic arbors and light scattering in the tissue (Ayling et al., 2009). A red-shifted ChR2 (Zhang et al., 2008), may be an advantage as red light will not scatter as much as blue light in the tissue. Another limiting factor for ChR2-mediated LBM is the limited availability of animal models, other than rodents, that express ChR2 over wide regions of cortex. Thus far, LBM has only been applied in the mouse (Ayling et al., 2009; Hira et al., 2009, 2013; Komiyama et al., 2010; Harrison et al., 2012) and the spatial resolution of LBM may be insufficient to map the finest details of the relatively small mouse motor cortex. Advancements in the generation of transgenic species, such as the rat (Tomita et al., 2009), or in ChR2 expression through *in utero* electroporation (Huber et al., 2008), would allow this technique to be applied to an animal model with a relatively larger motor cortex (Jazayeri et al., 2012), which may increase the method's relative resolution assuming factors such as light scattering are relatively constant across animals with variable motor cortex size (Aravanis et al., 2007). Furthermore, improvements in cell-specific ChR2 expression (Madisen et al., 2012) may allow for the generation of cell-specific maps within the sensorimotor cortex. Recently, we have applied LBM to investigate complex forelimb movements (Harrison et al., 2012). We found that prolonged stimulation of the sensorimotor cortex reliably evoked two types of forelimb movements—abduction and adduction movements—and these were generally organized within the sensorimotor cortex with the abduction movement representation anterior to the adduction movement representation. LBM has also been applied to identify tongue motor cortical areas for subsequent calcium imaging during a licking task in awake, head-fixed mice (Komiyama et al., 2010). Together, these studies demonstrate that LBM can be applied to map complex motor movements, and may allow us to draw conclusions about the organization of the sensorimotor cortex. In the future, LBM may be useful for the investigation of cortical map dynamics over time in a disease model (Carmichael, 2003; Dancause and Nudo, 2011), or after motor learning (Kleim et al., 1998).

### Functional regional mapping using optogenetic stimulation *in vivo*

Light-based mapping is an effective method for determining the organization of the sensorimotor cortex, yet because it is based on motor output rather than cortical output, it is limited to the sensorimotor cortex. Questions remain about the cortical connections and their function, such as: what are the cortical connections to and from a particular region of interest? Are other cortical regions involved in downstream processing? In order to answer these questions, a method capable of addressing the wide spatial scope of cortical activity is necessary.

Voltage sensitive dye (VSD) imaging is one method for investigating large-scale cortical ensemble activity and long-range connections *in vivo* (Kleinfeld and Delaney, 1996; Petersen et al., 2003; Grinvald and Hildesheim, 2004; Peterka et al., 2011). VSDs are membrane-bound, organic, small molecules that are used to monitor membrane potential changes through various biophysical mechanisms (for a full review, see Peterka et al., 2011), with the simplest mechanism being redistribution—the VSD incorporates into the cell membrane and changes in membrane potential cause the dye molecule to move into or out of the membrane, leading to relatively linear changes in the fluorescence or absorbance of the dye (Konnerth and Orkand, 1986; Shoham et al., 1999). VSD imaging has potentially diffraction-limited spatial resolution (up to 0.5 μm, Grinvald and Hildesheim, 2004) and high temporal resolution (on the order of microseconds; Grinvald and Hildesheim, 2004). Compared to some protein-based sensors, which depend on slow conformational changes of the protein to reflect change (Sakai et al., 2001; Knopfel, 2012), the effects of changing electrical field within the membrane are rapidly reflected in VSD fluorescence. Furthermore, VSD imaging does not filter for activity based on spiking, but reports membrane potential changes, so it reflects supra- and subthreshold neuronal activity (Berger et al., 2007). However, VSD imaging is limited because it stains all cells and all components of the membrane without discrimination (Chemla and Chavane, 2010), so these components cannot be separated within the VSD signal. Additionally, while intracellular microelectrode experiments (Konnerth and Orkand, 1986) and whole-cell voltage recordings (Petersen et al., 2003) have shown that VSD does not affect the intrinsic properties of the cell, there is evidence that prolonged illumination of the dye may cause photodamage and/or photobleaching (Scanziani and Hausser, 2009), making VSDs unsuitable for longitudinal studies. There have also been reports of VSD affecting GABAa receptor function (Mennerick et al., 2010; Grandy et al., 2012), albeit of conflicting sign. Nonetheless, the high spatial and temporal resolution of VSD imaging makes it an ideal method for mapping cortical function. In particular, red-shifted RH-series VSDs can be excited with long wavelength light (Grinvald and Hildesheim, 2004) distinct from the ChR2 activation spectrum (Airan et al., 2007; Zhang et al., 2010), making them an obvious choice for connectivity mapping (Wang et al., 2007; Lim et al., 2012).

VSD imaging has been employed to investigate long-range cortical connections and the propagation of cortical activity *in vivo* (Ferezou et al., 2007; Aronoff et al., 2010; Mohajerani et al., 2010), and it has been used to measure large-scale changes in cortical activity following brain injury such as stroke (Brown et al., 2009), or following sensory deprivation in the barrel cortex (Wallace and Sakmann, 2008). While structural connectivity can identify long-range connections (Miyashita et al., 1994), such as the connection between the barrel cortex and motor cortex (Hooks et al., 2011; Mao et al., 2011), VSD imaging can be used to further investigate the spatiotemporal dynamics of the connection (Ferezou et al., 2007). Using a combination of anatomical tracing methods and VSD imaging, Ferezou et al. (2007) confirmed the connection between barrel cortex and motor cortex, and found that the stimulation of the whisker or direct stimulation of the barrel cortex caused a subsequent VSD response in primary motor cortex within 8 ms, which is consistent with early *in vivo* work by Kleinfeld and Delaney, who first reported “satellite regions” of activity (Kleinfeld and Delaney, 1996). This response time is further consistent with anatomical tracing which suggests a monosynaptic connection between these two areas (Ferezou et al., 2007) and demonstrates the complementary nature of functional and structural connectivity.

VSD imaging studies have been important in identifying and quantifying properties of long-range connections in the mouse brain (Aronoff et al., 2010), yet they have been restricted to sensory cortical areas that can be activated through peripheral stimulation. Questions remain regarding the connectivity of secondary sensory cortices and association areas: what role do these areas have in long-range processing? Are input and output connections equal in strength between two cortical areas or is there a directionality to the connection? To answer these questions, a technique that could combine VSD imaging for recording of large-scale cortical activity with stimulation of multiple arbitrary cortical regions would be desirable. We have recently developed a method to address these questions using a combination of **VSD imaging and ChR2 stimulation** *in vivo* in the mouse (Lim et al., 2012). This allowed us to probe the cortex with high spatial and temporal precision, and investigate areas of the cortex that are not easily accessible through peripheral stimulation, such as association areas and secondary sensory areas (Figure 1D).

We used transgenic mice (line 18, from Jackson labs) that predominantly express ChR2 in layer 5 pyramidal neurons (Arenkiel et al., 2007). Mice were given a large craniotomy and the VSD response was recorded across the entire hemisphere (Lim et al., 2012). Using galvanometer mirrors to steer a 473 nm laser to various cortical regions, we were able to activate any area of the cortex, including less-studied regions like the parietal association cortex or secondary cortices (Carvell and Simons, 1986, 1987). The VSD response was recorded across an entire hemisphere and the strength of the response at specific regions of interest was calculated to estimate the strength of the connections between various regions of interest (Lim et al., 2012). From this, we calculated the strength of connections between regions and created a network diagram to quantitatively display the strength of connections between regions. Network analysis (Bullmore and Sporns, 2009) revealed a number of interesting points: we identified regions that were highly connected to many other regions (“hub regions”), regions with few connections, and asymmetrical connection strength between regions. For example, the connections from primary to secondary sensory areas were significantly stronger than the reciprocal connections (from secondary to primary sensory areas), suggesting more driver connections in the bottom-up direction than in top-down (Sherman and Guillery, 1998). The role of thalamic feedback loops or subcortical contribution to the VSD signal remain unclear when using this method. Recent structural tracing studies using wide-scale reconstruction of single neurons in Thy-1 GFP mice (Gong et al., 2013), suggests that layer 5 pyramidal neurons in somatosensory cortex may make corticothalamic connections, however it has previously been suggested that layer 5 input to thalamic nuclei are feedforward corticothalamocortical connections that are parallel to corticocortical connections (Sherman, 2007; Theyel et al., 2009). Nonetheless, the conclusions drawn from this method should be regarded as a representation of neocortical connectivity that could be affected by corticothalamic relays or feedback loops. In the future, we plan to use this method in a stroke model to investigate both local and long-range connectivity changes within the lesioned and non-lesioned hemispheres (Mohajerani et al., 2011). This method will be especially useful to probe for diaschisis—loss of function at sites distant from the injury site—after stroke (Feeney and Baron, 1986; Dancause and Nudo, 2011).

The combination of VSD imaging and ChR2 stimulation has the advantage of high spatial and temporal resolution for stimulating and recording, and can be done quickly and relatively non-invasively compared to traditional cortical probing methods that rely on electrical stimulation. However, photostimulation may cause photobleaching of the dye and saturation of the image (Lim et al., 2012), making high-intensity or long trains of stimulation challenging with this method. Nonetheless, the unique power of this method is in the ability to stimulate and record from a virtually unlimited number of cortical sites, with the resolution limited only by detection resolution and precision of optogenetic stimulation. In the Thy-1 transgenic mouse, ChR2 is expressed in both axons and dendrites of large layer 5 pyramidal neurons (Wang et al., 2007), which have pronounced apical dendritic tufts in layers 1 and 2/3 (Thomson and Lamy, 2007). Our functional experiments imply that photostimulation primarily activates these apical dendrites (which are prominent at the pial surface), leading to orthodromic activation of projecting axons, since regional connectivity maps were blocked by glutamate receptor antagonists (Lim et al., 2012). Thus, compared to electrical stimulation, we suggest that ChR2 stimulation within Thy-1 mice results in a lower contribution of direct axonal excitation, reducing the possibility of antidromic action potentials (Figure 2), which could be misinterpreted as a remote synaptic connection when only an axonal-to-somatic link exists. If ChR2 expression was more concentrated in neurons of more superficial layers (i.e., layer 2/3) in a transgenic mouse line or after *in utero* electroporation, it could lead to more frequent, direct activation of axons within superficial lamina, increasing the probability of antidromic action potentials. For this reason, the subcellular distribution of ChR2 must be taken into consideration. Fortuitously, deep layer neurons within the Thy-1 line 18 mice may not form many axonal connections to the superficial layers, which is consistent with previous work that has shown a strong functional connection from layer 2/3 to layer 5, but not from layer 5 to layer 2/3 (Weiler et al., 2008; Hooks et al., 2011).

Even greater specificity for regional functional mapping could be achieved with the development of new *in vivo* probes such as voltage sensitive fluorescent proteins (VSFPs), which can be targeted to specific cell populations and provide signals with no contribution from glia cells, blood vessels, or nearby silent neurons (Sakai et al., 2001; Akemann et al., 2010; Mancuso et al., 2010). Several variants of VSFPs have been developed (Perron et al., 2012). In particular, second generation VSFPs (VSFP2) have been developed through a molecular fusion of a voltage-sensing domain with a fluorescent reporter protein (Perron et al., 2009a), and report membrane potential change based on the Fluorescence Resonance Energy Transfer (FRET) principle (Perron et al., 2009b). Similar to VSDs, VSFPs are capable of reading out supra- and subthreshold activity from a large cortical area (Akemann et al., 2010). The greatest advantage of these probes over VSDs is that they can be targeted to a specific cell population, eliminating background contribution to the fluorescent signal, thus providing a more specific readout of neuronal activity (Akemann et al., 2010). This feature will be crucial in distinguishing whether different neuronal subtypes within a circuit serve different functions (Peterka et al., 2011). However, VSFPs are limited by slow kinetics, with on-rates on the order of 10 s of milliseconds (Scanziani and Hausser, 2009), and genetic voltage sensors have only had limited application in *in vivo* studies where high levels of expression were needed (however, see Akemann et al., 2010). There will also be considerable spectral overlap between VSFP and ChR2 necessitating the use of red-shifted opsins for neuronal stimulation and VSFP mapping.

Another set of tools for precise, cell-specific targeting are genetically encoded calcium indicators (GECIs). Calcium imaging has been applied over a range of spatial scales, from single cell calcium dynamics (Helmchen et al., 1999; Winship and Murphy, 2008; Golshani et al., 2009), to wide-field calcium imaging of population activity (Homma et al., 2009; Minderer et al., 2012). This wide scope of applications makes calcium imaging an attractive method for addressing functional mapping questions (Mancuso et al., 2010). GECIs are developed through a fusion of a calcium-sensitive molecule to one or two fluorescent protein molecules (Looger and Griesbeck, 2012), and report changes in calcium dynamics. Compared to VSDs and VSFPs, calcium probes are less-sensitive to subthreshold activity and, at the cellular level, are more related to spiking activity (Tian et al., 2009). However, because GECI signals are based on the timescale of calcium dynamics, they have a relatively slow decay time course, which may make it challenging to detect single action potentials *in vivo* (Scanziani and Hausser, 2009). GECIs also have the advantage of being stable over long periods of time *in vivo* (Looger and Griesbeck, 2012), making them attractive for longitudinal studies (Minderer et al., 2012). In mouse models, expression of GECIs has generally been achieved through adeno-associated virus (AAV) viral vector injection producing a spread of only 1–2 mm, however, new transgenic lines provide a widespread expression of GECI throughout the cortex (Zariwala et al., 2011; Chen et al., 2012a), allowing for large-scale functional connectivity studies. Moreover, there have been efforts to improve the sensitivity, temporal dynamics, and stability within the GCaMP family, a class of GECIs that fuses calmodulin to a single fluorescent protein (Tian et al., 2009; Zhao et al., 2011; Akerboom et al., 2012; Chen et al., 2012b), and to create blue and red-shifted GECIs for multi-color calcium imaging in single cells (Zhao et al., 2011). Until now, the use of GECIs with optogenetic stimulation has been limited due to spectral overlap between ChR2 and green GECIs (Hires et al., 2008), because both absorb blue light. However, the recent development of a new red-shifted rhodopsin (i.e., C1V1; Yizhar et al., 2011b) allowed for high-resolution two-photon imaging of green fluorescent proteins with simultaneous two-photon photostimulation (Rickgauer and Tank, 2012). This demonstrates the ability to combine optogenetic stimulation with calcium imaging, and the possibility for cell-specific probing of evoked calcium dynamics.

### Functional global mapping using optogenetic stimulation *in vivo*

Thus far, the methods described are effective for determining synaptic or even regional connectivity within the cortex, however, they are not capable of answering questions about global connectivity and the contribution of subcortical structures. Blood oxygenation level-dependent (BOLD) functional magnetic resonance imaging (fMRI) is a widely used technique for non-invasive, whole-brain imaging in animal and human studies. Although the relationship between BOLD signals and neuronal coupling is still incompletely understood (Logothetis and Pfeuffer, 2004; Logothetis, 2007), fMRI is an attractive technique for investigating the distribution of neuronal activity within the brain because it is non-invasive. Recently, fMRI has been combined with optogenetics (Desai et al., 2010; Lee et al., 2010; Kahn et al., 2011; Abe et al., 2012) to study the distribution of cortical and subcortical activity following cell-type-specific stimulation (Figure 1E). These studies demonstrated that **optogenetic fMRI (opto-fMRI)** could be used to stimulate network activity and identify long-range projections on a global scale.

The first study to employ opto-fMRI was by Lee et al. (2010). They used a rat model expressing ChR2 in the excitatory neurons of the motor cortex (M1) and observed activity in the thalamus after light stimulation of ChR2-expressing neurons in M1. Light pulses delivered via an optical fiber were able to reliably drive neuronal firing in ChR2-expressing neurons and in areas distal to the stimulation site, suggesting connectivity between the stimulation site and these distant areas. They further explored global cortical connections through optogenetic stimulation of different thalamic nuclei. Optogenetic stimulation of the posterior thalamic nucleus resulted in a BOLD response in the ipsilateral somatosensory cortex, while stimulation of the anterior thalamic nucleus resulted in a bilateral motor cortex BOLD response, suggesting that thalamic projections differ between the somatosensory cortex and the motor cortex, consistent with previous neuronal tracing reports (Alloway et al., 2008).

Opto-fMRI has also been applied in transgenic mice expressing ChR2 primarily in the layer 5 pyramidal neurons. Here, both the BOLD signal and the local field potential (LFP) responses to photostimulation were recorded (Lee et al., 2010; Kahn et al., 2011). ChR2 stimulation in the barrel cortex was shown to elicit similar regions of BOLD activity compared to sensory stimulation of the whiskers (Kahn et al., 2011), suggesting optogenetic stimulation is a feasible alternative for eliciting BOLD responses and determining global functional connections. In an awake mouse model, opto-fMRI was used to show connectivity between the primary barrel cortex (BCS1) and structures that are known to be connected to the primary somatosensory cortex, including secondary sensory areas and subcortical areas such as the striatum (Desai et al., 2010). ChR2 stimulation was able to reliably elicit a hemodynamic response (BOLD signal) in the local region of stimulation as well as downstream areas, consistent with BOLD fluctuations observed in human studies (Fox and Raichle, 2007). This correlation, combined with the ability to use opto-fMRI in an awake animal model, will make opto-fMRI an important technique for translational studies of disease, learning, or plasticity. However, because of technical constraints, it is not yet possible to produce multi-site high definition photostimulation as presented for other optogenetic mapping techniques (see above).

Opto-fMRI is limited by the incomplete understanding of the relationship between neural activity and cerebral blood flow. Previous reports have demonstrated that ChR2 activation in an *in vivo* mouse model results in increases in blood flow and oxygenation that can occur independent of ionotropic glutamatergic synaptic transmission (Scott and Murphy, 2012), presumably due to direct activation of neurons and secondary non-synaptic activation of other cells. Indeed, Lee et al. caution that the ChR2-evoked BOLD response may include contributions from cells other than those optically stimulated (Lee et al., 2010), as is likely the case for any active network of cells. Furthermore, a recent study suggests that light stimulation may effect the BOLD signal even in a naïve brain (Christie et al., 2012). Despite this incomplete understanding, some conclusions from opto-fMRI may be supported through additional measures, such as structural mapping (Assaf and Pasternak, 2008; Damoiseaux and Greicius, 2009) and due to the relatively non-invasive nature of opto-fMRI it remains a valuable platform for translational studies involving brain connectivity.

## Future prospects

In order to have a better understanding of the functional connectivity of the brain, several levels of organization need to be considered: synapses, circuits, systems, and whole-brain (Alivisatos et al., 2012). Furthermore, several brain states need to be considered, including anesthetized, awake, and awake and freely moving animals (Harris and Thiele, 2011). Future studies may also consider the developing brain, the injured brain, and learning and plasticity models to further enhance our understanding of brain function and dysfunction. The techniques reviewed here could be used to deduce impaired functional relationships between various cortical areas in disease models, especially for diseases such as autism where the underlying mechanism is poorly understood (Qiu et al., 2011), or could be used to examine and monitor recovery after brain injury, such as stroke (Murphy and Corbett, 2009; Krakauer et al., 2012). It is even possible that neuronal connectivity maps will be someday paired with maps of the cerebrovasculature (Tsai, 2009), to understand the effects of perturbations to flow during even relatively small strokes (Shih et al., 2013). Clearly, developing comprehensive maps is going to be a significant task due to the complexity of the brain and the number of variables that need to be considered. While there has yet to be developed a single perfect method for *in vivo* arbitrary point functional mapping (Table 2), through combining information gathered from a variety of techniques and by taking advantage of technological advancements such as optogenetics, we will continue to gain important insights on cortical organization and function.

**Table 2:** **Properties of an ideal functional mapping technique**.

**Property**	**Description**	**Suitable techniques**	**Unsuitable techniques**
Longitudinal assessment	Relatively non-invasive and non-toxic for longitudinal (chronic) studies	LBM	VSD-ChR2
		VSFPs	CRACM
		GECI-C1V1	
		Opto-fMRI	
High temporal resolution	Relatively fast kinetics (on the order of milliseconds); on par with action potentials	CRACM	Opto-fMRI
		LBM	
		VSD-ChR2	
		GECI-C1V1	
Wide spatial scale	Can map activity over a large field of view; can track long-range projections	VSD-ChR2	CRACM
		Opto-fMRI	LBM
Arbitrary point stimulation	Can stimulate defined sites in rapid succession	CRACM	Opto-fMRI
		LBM	
		VSD-ChR2	
Reports neuronal activity	Reports membrane potential changes	CRACM	LBM
		VSD-ChR2	Opto-fMRI
		GECI-C1V1	
Cell-specific reporting	Reports activity from a defined cell population	CRACM	VSD-ChR2
		GECI-C1V1	Opto-fMRI

### Conflict of interest statement

The authors declare that the research was conducted in the absence of any commercial or financial relationships that could be construed as a potential conflict of interest.

## Key Concept

ChR2-assisted circuit mapping (CRACM): A method for *in vitro* functional circuit mapping based on laser scanning photostimulation (LSPS), where the connections between presynaptic ChR2-expressing cells and postsynaptic neurons are mapped using whole-cell recordings (Petreanu et al., 2007).
Light-based mapping (LBM): A method for *in vivo* functional mapping of the sensorimotor cortex, based on ChR2-evoked motor responses. To determine cortical function and organization, motor movements (electromyograms) are recorded following targeted photostimulation of ChR2-expressing cells in the motor cortex (Ayling et al., 2009; Hira et al., 2009).
VSD imaging and ChR2 stimulation: A method for large-scale *in vivo* functional mapping of the cortex based on VSD imaging with RH1692 combined with ChR2 stimulation. Connections between cortical regions are mapped by targeting multiple cortical regions for ChR2 photostimulation and recording subsequent changes in VSD fluorescence at multiple cortical regions (Lim et al., 2012).
Optogenetic fMRI (opto-fMRI): A method for *in vivo* functional mapping of the brain, where connections between ChR2-expressing cells and their long-range projections are mapped using single-point optogenetic stimulation and subsequent BOLD signal responses in the brain (Desai et al., 2010; Lee et al., 2010).
